# Influence of upper blepharoplasty on intraocular lens calculation

**DOI:** 10.5935/0004-2749.20210002

**Published:** 2025-02-02

**Authors:** Maria Eugenia Vola, Renato Lisboa, Evandro Ribeiro Diniz, Nicolas Cesário Pereira, Ricardo Tomoyoshi Kanecadan, Adriana dos Santos Forseto

**Affiliations:** 1 Hospital Oftalmológico de Sorocaba, Sorocaba, Brazil; 2 Botelho Hospital da Visão, Blumenau, Brazil; 3 Ciências Médicas de Minas Gerais, Belo Horizonte, Brazil; 4 Eye Clinic Day Hospital, São Paulo, Brazil

**Keywords:** Blepharoplasty, Intraocular lens, Keratometry, Corneal topography, Biometry, Blefaroplastia, Lentes intraoculares, Ceratometria, Topografia da córnea, Biometria

## Abstract

**Purpose:**

To determine the effect of upper blepharoplasty on corneal topography and intraocular lens power calculation using Galilei and IOLMaster.

**Methods:**

Thirty patients submitted to upper blepharoplasty from May 2014 to March 2017 at the Hospital Oftalmológico de Sorocaba (São Paulo, Brazil) were included in this observational case series. All patients underwent imaging sessions with Galilei and IOLMaster preoperatively (baseline) and at 1 and 6 months postoperatively. Primary outcome measures using both devices included flattest, average, and steepest corneal curvature, corneal astigmatism, and blepharoplasty-induced corneal astigmatism. Determination of axial length and lens power calculation were performed using only IOLMaster (Holladay formula). Paired t-test and vectorial analysis were used for statistical analysis.

**Results:**

Sixty eyes from 30 patients were prospectively included. Vectorial analysis showed that 6 months after surgery, blepharoplasty induced on average 0.39 D and 0.31 D of corneal astigmatism, as measured with Galilei and IOLMaster, respectively. IOLMaster measurements showed that average corneal curvature (44.56 vs 44.64 D, p=0.01), steepest corneal curvature (45.17 vs 45.31, p=0.01) and corneal astigmatism (1.22 vs 1.34, p=0.03) were higher 6 months after surgery. IOLMaster measurements also showed that intraocular lens power was significantly smaller 6 months after surgery (22.07 vs 21.93, p=0.004). All other parameters showed no change for comparisons between baseline and 6 months (p>0.05 for all comparisons).

**Conclusion:**

Upper eyelid blepharoplasty influenced intraocular lens calculation using the IOLMaster. However, the influence was not clinically significant. No topographic changes were found using Galilei.

## Abstract (pt)

**Objetivo:**

Determinar o efeito da blefaroplastia superior na topografia corneana e no cálculo do poder das lentes intraoculares usando Galilei e IOLMaster.

**Métodos:**

Trinta pacientes submetidos a blefaroplastia superior de maio de 2014 a março de 2017 no Hospital Oftalmológico de Sorocaba, São Paulo, Brasil foram incluídos neste estudo de série de casos observacional. Todos os pacientes foram submetidos a sessões de imagem com Galilei e IOLMaster antes da cirurgia (exame de base) e no 1º e 6º mês pós-operatório. Os resultados primários utilizando os dois aparelhos incluíram ceratometria, astigmatismo corenano e astigmatismo corneano induzido pela blefaroplastia. O comprimento axial e o cálculo do poder da lente intraocular foram realizados unicamente com o IOLMaster (fórmula de Holladay). Teste-t pareado e análise vetorial foram usados na análise estatística.

**Resultados:**

Sessenta olhos de 30 pacientes foram incluídos prospectivamente. A análise vectorial mostrou que após 6 meses da cirurgia, a blefaroplastia superior induziu na média 0,39 D de astigmatismo corneano medido com o Galilei e 0,31 D com IOLMaster. As medidas com o IOLMaster mostraram que a ceratometria média (44,56 vs 44,64 D, p=0,01), ceratometria máxima (45,17 vs 45,31, p=0,01) e o astigmatismo corneano (1,22 vs 1,34, p=0,03) foram maiores após 6 meses da blefaroplastia. As medidas com IOLMaster mostraram que o poder da lente intraocular foi significativamente menor 6 meses após a blefaroplastia (22,07 vs 21,93, p=0,004). Todos os outros parâmetros não mostraram mudanças entre o pré-operatório e o 6º mês da cirurgia (p>0,05 para todas as comparações).

**Conclusões:**

A blefaroplastia superior influenciou o cálculo da lente intraocular utilizando o IOLMaster. Contudo, a influência não foi clin icamente significativa. Não foram encontradas mudanças topográficas com o Galilei.

## INTRODUCTION

Dermatochalasis is an age-related condition, characterized by excessive skin at the upper eyelid, which can only be treated with surgery (i.e., upper blepharoplasty). Cataract is more frequent in the elderly and also only treated with surgery. Upper blepharoplasty and cataract surgery are two of the most commonly performed procedures in ophthalmology^(1,2)^. The timing of the procedures is an important concern for the ophthalmologist, because the pressure exerted by the superior eyelid can affect the corneal curvature and therefore influence the intraocular lens (IOL) power calculation for cataract surgery^(3)^.

IOL power calculation has gained great interest in the era of refractive cataract surgery. In recent years, a growing number of devices have been developed to measure the clinical parameters necessary for the calculation of the IOL power^(4)^. These parameters include the flattest, average, and steepest corneal curvature, as well as the axial length.

Although previous studies have evaluated the changes on corneal curvature following blepharoplasty, this is the first study to use the Galilei and IOLMaster devices for this purpose. It is also the first study that evaluated the induced corneal astigmatism after superior blepharoplasty using vectorial analysis.

## METHODS

This observational case series study, conducted from May 2014 to March 2017, included patients from the oculoplastic clinic at the Hospital Oftalmológico de Sorocaba (São Paulo, Brazil). Informed consent was obtained from all participants and all protocols were approved by the Hospital Ethics Committee. Methods attended to the tenets of the Declaration of Helsinki.

All patients underwent comprehensive ocular examination, including best-corrected visual acuity, ocular pressure measurement using Goldmann applanation tonometry, anterior biomicroscopy, and fundus examination at the preoperative visit (baseline). Moreover, at baseline and follow-up (1 and 6 months), patients underwent ancillary examinations with Galilei Dual Scheimpflug Analyzer G4 (Ziemer, Switzerland) and ocular biometry with IOLMaster 500 (Carl Zeiss Meditec Inc., Dublin, CA, USA). Inclusion criteria were best-corrected visual acuity of ≥20/40, ametropia <6 D, and dermatochalasis. Patients with coexisting corneal disease, macular pathology, glaucoma, uveitis, ptosis, and/or history of previous ocular or palpebral surgery were excluded.

Thirty patients scheduled for upper eyelid blepharoplasty were recruited from the oculoplastic clinic. Surgery was performed by residents or fellows using the same technique. After marking, the excessive skin was removed using a blade and scissors. If a prominent fat pad was present it was removed using cautery and scissors. The skin was sutured with 6-0 nylon sutures that were removed 1 week after surgery. To be included in the study patients had to attend to all visits.

### Ancillary exams

The Galilei is a non-invasive device designed for the analysis of the anterior segment of the eye. It is based on a rotating dual-Scheimpflug camera integrated with a Placido topographer. This device captures slit images from opposite sides of the illuminated slit and averages the elevation data obtained from corresponding opposite slit images. The following corneal parameters provided by the Galilei were included and analyzed in our study: corneal curvature (flattest, average, and steepest) and corneal astigmatism (i.e., arithmetical difference between the flattest and steepest corneal curvatures). Anterior chamber depth (ACD) and axial length were not available for us. Therefore, Galilei was not used as a biometer.

The IOLMaster is an optical biometry device that emits infrared light at 780 nm and uses partial coherence interferometry to measure the ACD and axial length. For measurements of ACD, it uses a 0.7-mm-wide slit beam of light directed at a 30° angle into the anterior chamber. Subsequently, it measures the distance between the light reflection on the anterior corneal surface and the anterior crystalline lens surface. The device uses an average of five serial measurements along the visual axis to determine the final ACD value^(5)^.

The IOLMaster also provides measurements of the corneal curvature. Together, these parameters are used to calculate the IOL power. Parameters provided by the IOLMaster and analyzed in our study included flattest corneal curvature, average corneal curvature, steepest corneal curvature, corneal astigmatism, axial length, and IOL power calculation using Holladay’s formula for emmetropia. For a better description of the results, we divided the IOL power changes in three groups: 1) changes ≤0.5 D; 2) changes >0.5 and ≤1 D; 3) changes >1 and ≤2 D

### Statistical analysis

We performed a sample size analysis. Alpha (type I error) and beta (type II error) levels were set at 0.05 and 0.2, respectively. Effect size was set at 0.2. A conservative standard deviation of the outcome (in our case, the difference between IOL powers at 6-month follow-up and baseline) of 0.75 was used. Within-subject correlation of the outcome was set at 0.875. The number of eyes was estimated as 28^(6)^.

**Figure 1:**
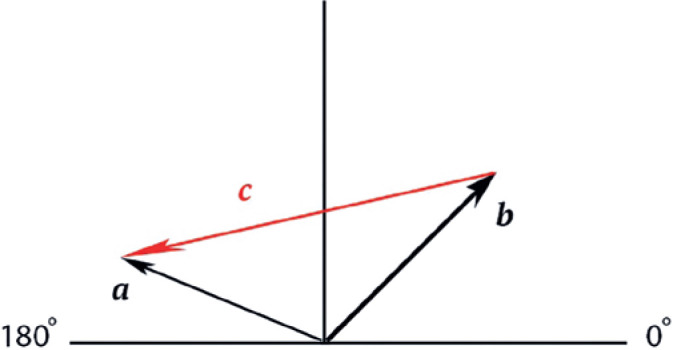
Diagram representing the relationship between preoperative astigmatism (***a***), postoperative astigmatism (***b***), and blepharoplasty-induced astigmatism (***c***).

Vectorial analysis was performed to evaluate the magnitude and axis of the blepharoplasty-induced corneal astigmatism. This approach for the study of the corneal astigmatism was first described by Alpins et al., in patients subjected to refractive surgery^(7)^. In our study, blepharoplasty-induced astigmatism was defined as the vectorial difference between the corneal preoperative and postoperative astigmatism (Figure 1). The following are the definitions used in this study:

***a*** = preoperative astigmatism vector
***b*** = postoperative astigmatism vector
***c*** = blepharoplasty-induced astigmatism

As shown in figure 1, the vector **c** can be mathematically defined as


c=b-a

Any vector *v* can be described by its coordinates (x, y) in a Cartesian plane. In this study, the magnitude of the vector *c* was calculated using the following formula:


$$c=\left|\frac{y_{b}-y_{a}}{\sin\left\{\tanh^{-1}\left[\frac{\left(y_{b}-y_{a}\right)}{\left(x_{b}-x_{a}\right)}\right]\times 180/\pi \right\}}\right|$$

The coordinates x and y can be determined by the following formulas, where *m* and *n* represent the magnitude and the axis of any astigmatism vector *v*:


$$\begin{array}{rl} & x=m\times \cos(2\times n\times \pi /180)\\ & y=m\times \sin(2\times n\times \pi /180) \end{array}$$

Finally, the axis of the vector *c* can be determined by the following formula:


$$\text{ axis }=\left\{\tanh^{-1}\left[\left(y_{b}-y_{a}\right),\left(x_{b}-x_{a}\right)\right]\times 180/\pi \right\}+90$$

Descriptive statistics included mean, standard deviation, 25^th^ and 75^th^ percentiles. In order to compare the studied parameters between visits a paired t-test was used. To account for potential correlation between eyes, the cluster of data for the studied subject were considered as the unit of resampling when calculating standard errors. This procedure has been used to adjust for the presence of multiple correlated measures of the same unit^(8)^. Specifically, in the ophthalmic literature, this procedure has been used to adjust for the presence of both eyes of the same patient in the study^(9)^.

Statistical analyses were performed with Stata (version 12, StataCorp, College Station, TX, USA) and Microsoft Excel (version 16.12; Microsoft, Redmond, WA, USA) software. The alpha level (type I error) was set at 0.05.

## RESULTS

A total of 60 eyes from 30 patients (27 females and 3 males), aged 47.7-74.4 years (mean: 58.5 years ± 6.9 years) were included in the study.

Table 1 shows the mean values (± standard deviation, 25^th^ and 75^th^ percentiles) of blepharoplasty-induced corneal astigmatism after 1 and 6 months using vectorial analysis. At 6 months, upper blepharoplasty induced on average 0.39 D of corneal astigmatism, as measured with Galilei and 0.31 D as measured with IOLMaster. After 6 months, 51 eyes had an IOL power change ≤0.5 D, 8 eyes had an IOL power change >0.5 and ≤1 D and only 1 eye had an IOL power change >1 and ≤2 D.

**Table 1:** Mean ± SD (25^th^ and 75^th^ percentiles) of blepharoplasty induced corneal astigmatism 1 and 6 months after surgery measured with Galilei and IOLMaster using vectorial analysis.

		1 month		6 months
	**BIA (diopters)**	**BIA (degrees)**	**BIA (diopters)**	**BIA (degrees)**
Galilei	0.41 ± 0.29 (0.17, 0.56)	81.32 ± 52.40 (32.92, 126.84)	0.39 ± 0.31(0.18, 0.56)	85.69 ± 48.84 (44.50, 130.00)
IOLMaster	0.43 ± 0.28 (0.22, 0.56)	101.56 ± 53.98 (59.74, 144.30)	0.31 ± 0.32 (0.09, 0.41)	89.70 ± 52.05 (41.00, 134.00)

BIA= Blepharoplasty-induced astigmatism; SD = Standard deviation.

**Figure 2:**
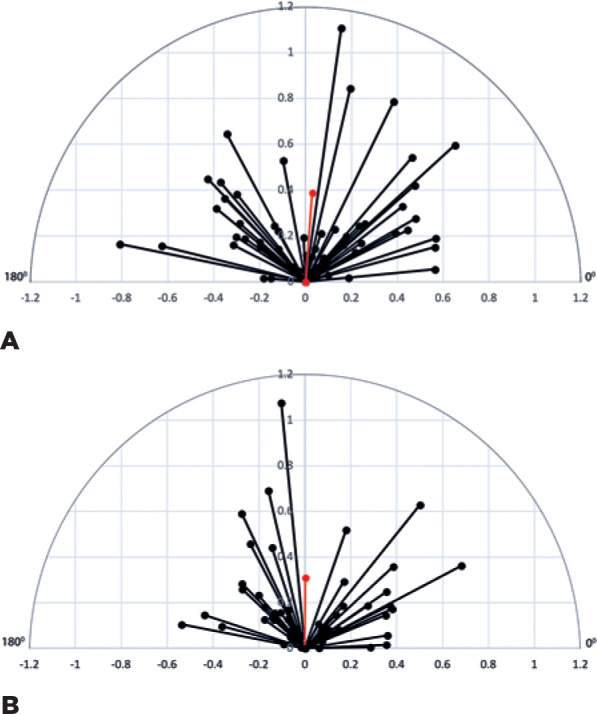
A) Vectorial display of induced corneal astigmatism 6 months after surgery measured with the Galilei. Vector mean (0.39 D at 85.69°) is represented in red. B) Vectorial display of induced corneal astigmatism 6 months after surgery measured with the IOLMaster. Vector mean (0.31 D at 89.70°) is represented in red.

Figure 2A shows the vectorial display of induced corneal astigmatism 6 months after blepharoplasty measured with the Galilei. Figure 2B shows the vectorial display of induced corneal astigmatism 6 months after blepharoplasty measured with the IOLMaster. Vectors means on figures 2A and 2B are represented in red.

Table 2 shows mean values of Galilei and IOLMaster parameters at baseline and follow-up (1 and 6 months). Comparisons between baseline and 1-month parameters showed no change with both devices (p>0.05 for all comparisons). Comparison between baseline and 6 months showed differences for four parameters provided by the IOLMaster. Average corneal curvature (44.56 vs 44.64, p=0.01), steepest corneal curvature (45.17 vs 45.31, p=0.01) and corneal astigmatism (1.22 vs 1.34, p=0.03) were higher after 6 months. IOL power was significantly smaller (22.07 vs 21.97, p=0.004) after 6 months of upper blepharoplasty. All other parameters showed no change for comparisons between baseline and 6 months (p>0.05 for all comparisons).

**Table 2:** Mean values ± SD (25^th^ and 75^th^ percentiles) of Galilei and IOLMaster parameters during the follow-up (for baseline, 1 month and 6 months of follow-up).

Parameter	Baseline	1 month	6 months	p-value for baseline vs 1 month	p-value for baseline vs 6 months
**Galilei**					
Flattest corneal curvature (diopters)	44.05 ± 1.27(43.06, 45.00)	44.03 ± 1.27(43.00, 44.77)	44.07 ± 1.29 (43.15, 45.16)	0.68	0.61
Average corneal curvature (diopters)	44.65 ± 1.21 (43.95, 45.52)	44.65 ± 1.24 (43.86, 45.33)	44.69 ± 1.24 (43.86, 45.57)	0.93	0.29
Steepest corneal curvature (diopters)	45.26 ± 1.39 (44.20, 46.23)	45.27 ± 1.42 (44.33,46.22)	45.30 ± 1.42 (44.38, 46.28)	0.80	0.19
Corneal astigmatism (diopters)	1.20 ± 1.12 (0.49, 1.45)	1.24 ± 1.05 (0.55, 1.42)	1.22 ± 1.10(0.51, 1.35)	0.54	0.66
Corneal astigmatism axis (degrees)	91.83 ± 35.85 (72.50, 104.50)	90.60 ± 41.39 (73.50, 109.00)	93.48 ± 39.72 (74.50, 105.00)	0.82	0.54
**lOLMaster**					
Flattest corneal curvature (diopters)	43.95 ± 1.30 (42.99, 44.94)	43.94 ± 1.30 (42.88, 44.91)	43.96 ± 1.29 (43.02, 44.88)	0.81	0.49
Average corneal curvature (diopters)	44.56 ± 1.26 (43.73, 45.39)	44.60 ± 1.26 (43.82,45.44)	44.64 ± 1.25 (43.92, 45.32)	0.17	0.01
Steepest corneal curvature (diopters)	45.17 ± 1.46 (41.14, 45.98)	45.25 ± 1.44 (44.26, 46.11)	45.31 ± 1.47 (44.41,46.26)	0.07	0.01
Corneal astigmatism (diopters)	1.22 ± 1.14 (0.47, 1.33)	1.31 ± 1.10 (0.61, 1.40)	1.34 ± 1.16 (0.52, 1.52)	0.08	0.03
Corneal astigmatism axis (degrees)	98.51 ± 65.87(21.50, 162.50)	93.50 ± 66.41 (10.50, 161.00)	101.6 ± 65.47 (37.50, 165.50)	0.54	0.71
Axial length (millimeters)	22.92 ± 0.76 (22.43, 23.55)	22.92 ± 0.76 (22.44, 23.55)	22.92 ± 0.76 (22.45, 23.54)	0.45	0.13
Intraocular lens power (diopters)	22.07 ± 2.09 (20.08, 23.54)	22.00 ± 2.06 (20.81,23.34)	21.93 ± 2.10(20.64, 23.39)	0.08	0.004

SD= standard deviation.

**Figure 3:**
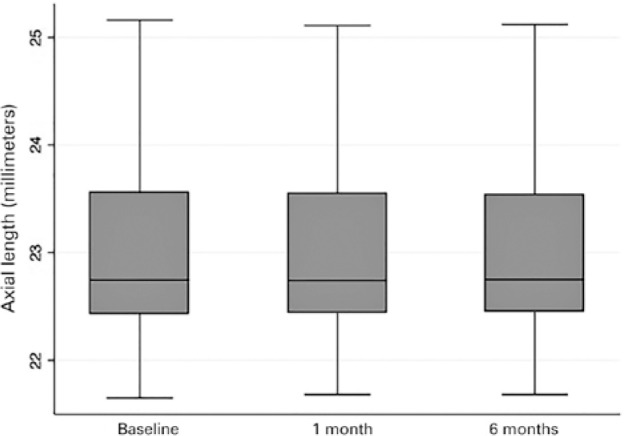
Box plot graphic for axial length at baseline and follow-up (1 and 6 months visits) measured with the IOLMaster.

**Figure 4:**
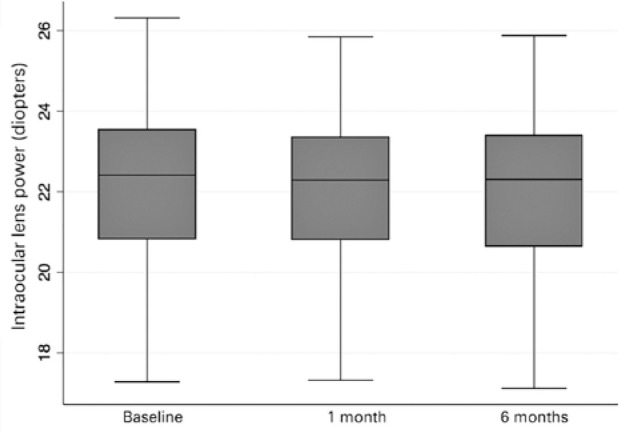
Box plot graphic for intraocular lens power at baseline and follow-up (1 and 6 months) provided by the IOLMaster.

Figures 3 and 4 show box plot graphics for axial length and IOL power at baseline and follow-up (1 and 6 months) provided by the IOLMaster.

## DISCUSSION

Corneal keratometry and axial length are parameters that have a large influence on IOL power calculation^(10)^. In our study, we analyzed the influence of upper blepharoplasty on these parameters and showed that this surgery influences IOL power calculation for cataract surgery. To the best of our knowledge, this is the first study to reach this conclusion using IOLMaster. Moreover, it is the first study to evaluate blepharoplasty-induced corneal astigmatism after upper blepharoplasty using vectorial analysis.

It is well established that the pressure of the upper eyelid on the cornea influences its shape^(3)^. Previous studies reported that eyelid surgery changes corneal curvature and that the extent of these changes is associated with more profound palpebral modifications. Removal of skin with blepharoplasty may lead to redistribution of the pressure applied by the lids over the cornea and consequently result in changes in the corneal shape and axial length measurements^(11-13)^.

Dogan et al. described in a study with 30 patients that repositioning the upper eyelid through blepharoplasty seems to cause steepening in the steepest corneal curvature only in patients with some degree of ptosis (i.e., superior margin reflex distance <2.5 mm). There were no topographic changes found in patients with a superior margin reflex >2.5 mm^(13)^. Upper eyelid height also has an influence on corneal curvature. Although repair of ptosis influences the corneal curvature, we excluded patients with ptosis in this study. Therefore, we did not take into consideration the eyelid height in our analysis^(14-17)^. We used this approach to avoid possible confounding factors between surgeries on the inter pretation of our results.

On the other hand, we cannot disregard the induced astigmatism calculated by the vectorial analysis. Minimal variations in corneal astigmatism may influence the indication of a toric IOL, especially for those who undergo implantation of bifocal or trifocal lenses^(18)^. Residual postoperative astigmatism after cataract surgery is an important source of visual complaints in these patients^(19)^.

Interestingly, statistically significant corneal changes were found only in IOLMaster measurements. Our findings may be explained by the different methodology used by Galilei and IOLMaster to measure the corneal curvature. Galilei uses Placido rings and a series of Scheimpflug images to measure the corneal curvature using data from 1 to 4 mm of the central cornea. Conversely, the IOLMaster measures corneal curvature by the reflection of projected points in the 2.50 mm central cornea. The instrument measures the distances between opposite points, securing three meridians, and calculates the corneal curvature. Lopez de La Fuente et al. had previously reported differences between Galilei and IOLMaster when measuring the corneal curvature^(20)^. As discussed by the author, although these differences can be statistically significant, they are probably not clinically significant.

Changes on corneal curvature following upper blepharoplasty were probably responsible for changes in IOL power between baseline and after 6 months (22.07 D vs. 21.93 D, p=0.004). Although a statistical decrease in IOL power was found in our study, this change was not clinically significant, as a 0.14 D difference on lens selection will not greatly influence the visual outcome after cataract surgery. However, it is important to highlight that nine of 60 eyes (15%) had a change in IOL power calculation >0.5 D, which may lead to patient dissatisfaction. Therefore, we suggest a personalized analysis in clinical practice, especially for patients with higher expectations and more severe dermatochalasis. In these cases, cataract surgery should be performed at least 6 months after palpebral surgery.

Our study had limitations. We did not perform IOL power calculations using the Galilei. According to the Galilei device, the corneal curvature remained unaltered after surgery. Thus, it was expected that the IOL power would also be similar after upper eyelid surgery. Moreover, differences in IOL calculations between Galilei and IOL Master in healthy patients were assessed by other authors and although minor differences have been reported they were not clinically significant^(21)^. We only used the Holladay formula to calculate the IOL power. Hence, further investigations are warranted to compare the results obtained using different formulas.

In conclusion, upper eyelid blepharoplasty influenced IOL power calculation for cataract surgery using the IOLMaster; however, this influence was not clinically significant. This change on IOL power is probably secondary to corneal curvature changes. Similar changes were not found in corneal tomographic parameters provided by the Galilei. Our findings are relevant due to the growing number of cataract and upper blepharoplasty surgeries performed on elderly patients.
